# Photo-acoustic spectroscopy revealing resonant absorption of self-assembled GaAs-based nanowires

**DOI:** 10.1038/s41598-017-02839-1

**Published:** 2017-06-06

**Authors:** Grigore Leahu, Emilija Petronijevic, Alessandro Belardini, Marco Centini, Roberto Li Voti, Teemu Hakkarainen, Eero Koivusalo, Mircea Guina, Concita Sibilia

**Affiliations:** 1 Dipartimento di Scienze di Base ed Applicate per l’Ingegneria, Sapienza Università di Roma, A. Scarpa 16, 00161 Rome, Italy; 2 Optoelectronics Research Centre, Tampere University of Technology, Korkeakoulunkatu 3, 33720 Tampere, Finland

## Abstract

III–V semiconductors nanowires (NW) have recently attracted a significant interest for their potential application in the development of high efficiency, highly-integrated photonic devices and in particular for the possibility to integrate direct bandgap materials with silicon-based devices. Here we report the absorbance properties of GaAs-AlGaAs-GaAs core-shell-supershell NWs using photo-acoustic spectroscopy (PAS) measurements in the spectral range from 300 nm to 1100 nm wavelengths. The NWs were fabricated by self-catalyzed growth on Si substrates and their dimensions (length ~5 μm, diameter ~140–150 nm) allow for the coupling of the incident light to the guided modes in near-infrared (IR) part of the spectrum. This coupling results in resonant absorption peaks in the visible and near IR clearly evidenced by PAS. The analysis reveal broadening of the resonant absorption peaks arising from the NW size distribution and the interaction with other NWs. The results show that the PAS technique, directly providing scattering independent absorption spectra, is a very useful tool for the characterization and investigation of vertical NWs as well as for the design of NW ensembles for photonic applications, such as Si-integrated light sources, solar cells, and wavelength dependent photodetectors.

## Introduction

The growing need for fast, integrated, nanoscale, low-cost photonic devices has triggered intensive developments of semiconductor nanowires (NWs). Main efforts have been directed to realize high quality nano-structures that are able to confine optical fields, and enhance the electrical and optical response in nano-scale dimensions. A single III-V semiconductor vertical NW has good waveguiding properties for energies above the bandgap owing to high refractive indices, and it can support a number of discrete photonic modes. These confined modes can lead to resonant absorption at specific wavelengths mainly defined by the combination of materials properties and their geometric dimensions^1^. The coupling of light to guided modes is essential for light harvesting and lasing applications; for example for the demonstration of NW lasing up to room temperature^2^, and monolithic integration of NW lasers on Si^3^. The resonant absorption in NWs has been studied numerically: for single NWs and NW arrays^4^, and for both parallel and normal excitation with respect to the long NW axis^5^. Recently, wavelength selective photodetectors^6^ and spin angular momentum generation^7^ based on the absorption enhancement have been proposed. Recent developments in fabrication of semiconductor NWs have already lead to a vast range of applications: photo voltaics^8–11^, light sources^12, 13^, waveguides^14^, photodetectors^15^ and water reduction^16^, all of which exploit the confining properties of the NWs. In particular, NW solar cells reaching efficiencies up to 15.3% have been recently demonstrated owing to resonant absorption enabling bulk-like photocurrent generation with greatly reduced material consumption^17^. Furthermore, NWs can exhibit nonlinear optics effects as second harmonic generation^18^ and finally, the hybridization of dielectric NWs with metallic layers and/or nanostructures offers a wide range of new application possibilities such as novel yagi-uda antennas^19^, and plasmonic lasers^20–22^.

Although the optical and electrical properties have been widely investigated and well understood, the characterization techniques are usually based on the indirect measurements of the resonant absorption by means of transmission/reflection^23^, photocurrents^24^ or photoluminescence excitation^25–27^. Recently, a scattering free photo-acoustic technique has been applied to determine the absorption edge of GaAsBi/GaAs NWs^28^. However, the absorption measurements that would provide the detection of the resonant NW behaviour and therefore direct detection of discrete waveguide modes are still lacking. In this work we used the photo-acoustic spectroscopy (PAS) technique to study absorbance properties of GaAs-AlGaAs heterostructure NWs grown by self-catalyzed technique on lithography-free Si/SiOx patterns^29^.

**Figure 1:**
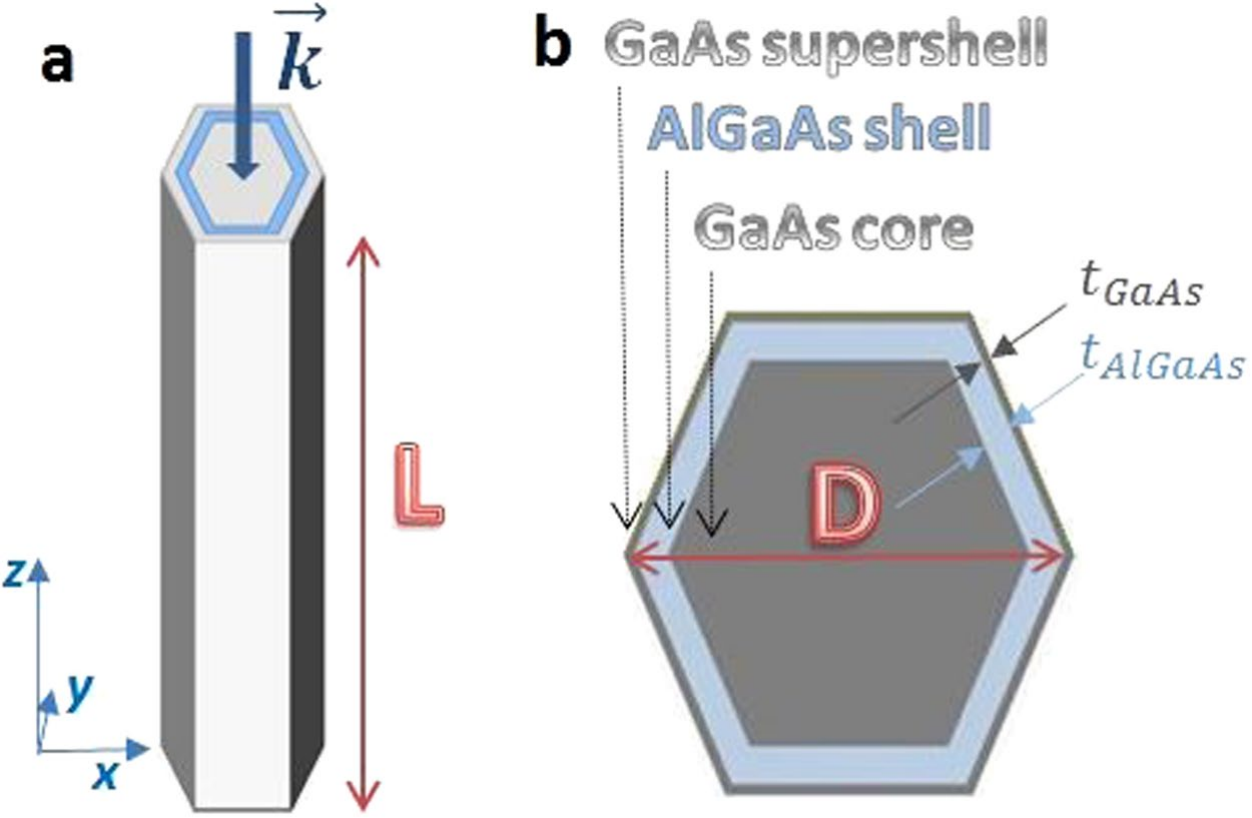
(**a**) Vertical schematic of the NWs of the length L, where $\vec{k}$ is always parallel to L and normally incident on the cross section. (**b**) Cross section of the NWs with overall diameter D, which comprises GaAs core, t_AlGaAs_ thick AlGaAs shell, and t_GaAs_ thick GaAs supershell.

The structures under examination are coaxial core-shell-supershell NWs with hexagonal cross section; schematic of a single NW is shown in Fig. 1a. NWs are made of GaAs core, surrounded by a thin shell of AlGaAs, around which there is a thin supershell of GaAs; the cross section is shown in Fig. 1b. Characteristic geometric parameters are the NW length L, the overall diameter D, AlGaAs shell thickness t_AlGaAs_, and GaAs supershell thickness t_GaAs_. We investigate four samples with different NW dimensions, the parameters of which are given in Table 1. In all work that follows we consider the light normally incident on the cross-section plane, where $\vec{k}$ is parallel to L.

**Table 1:** Characteristic geometric parameters for the four samples together with their standard deviations.

Sample	L [nm]	D [nm]	t_AlGaAs_ [nm]	t_GaAs_ [nm]
A	4750 ± 34	138 ± 5	3.5	0.7
B	5190 ± 64	151 ± 5	8.6	1.7
C	4600 ± 52	165 ± 6	11.7	5.8
D	4690 ± 47	197 ± 9	27.7	5.5

The four samples exhibit low fabrication error margins.

## Results

**Figure 2:**
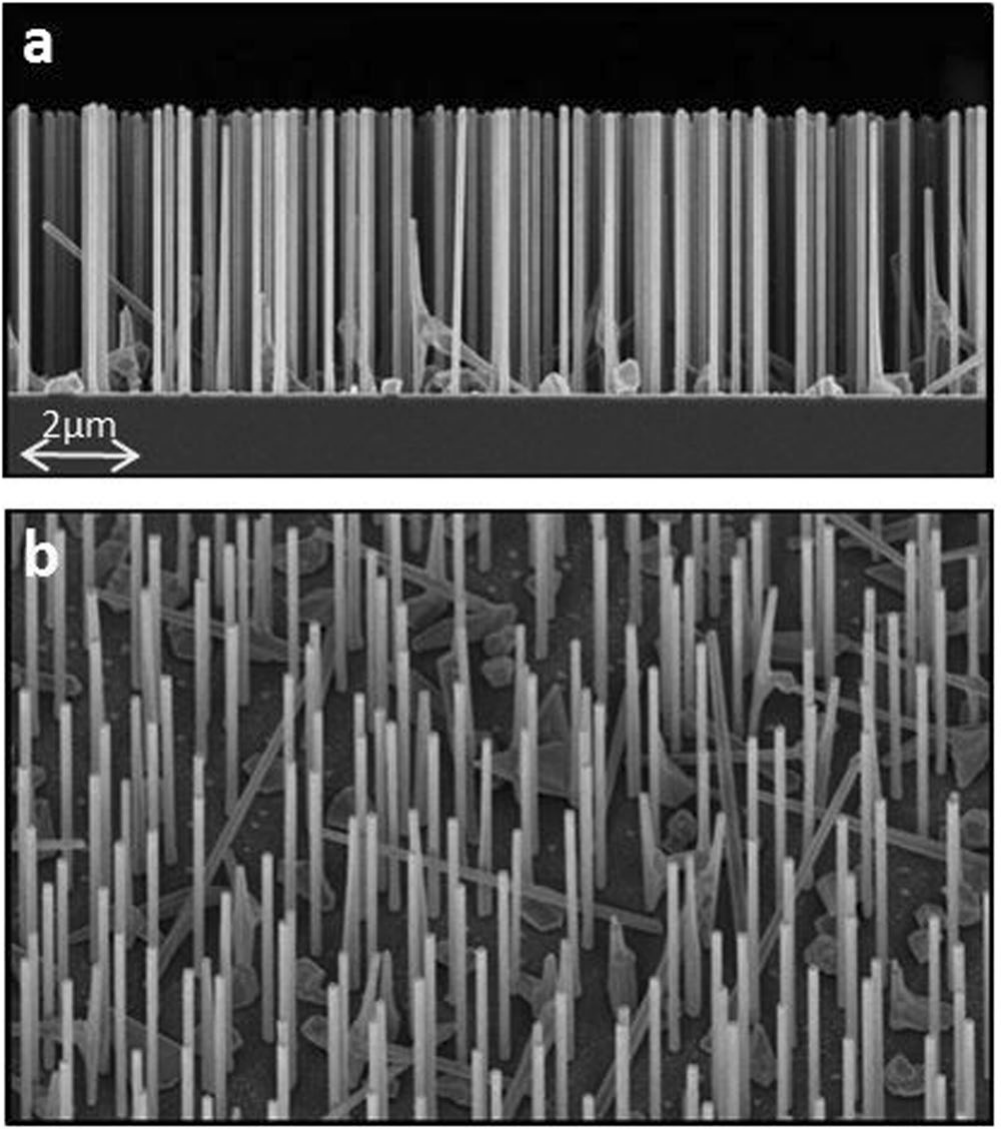
(**a**) SEM edge view of Sample A with NWs of L = (4750 ± 34)nm, D = (138 ± 5)nm. (**b**) Tilted (30°) SEM image of 3D distribution of Sample A.

The NWs were grown by molecular beam epitaxy on p-Si(111) wafers using lithography-free Si/SiOx patterns for defining the nucleation sites. This technique enables fabrication of highly uniform NW ensembles with tailorable NW density (see Methods). In Fig. 2 we show SEM images of the sample A to put into evidence their high quality and uniformity, that also applies to samples B, C and D (Supporting Information, Fig. S1). Since NW distribution does not show any periodical dependence, we can further assume that the response of this ensemble mainly depends on a single NW behaviour rather than on a collective response. We have then obtained the NW size statistics by measuring dimensions of more than 20 NWs per sample from cross-sectional SEM micrographs. The resulting statistics (Table 1) show that the fabrication deviations are small for L and D. The samples also differ slightly in the NW density: for samples A, C and D ρ_A,C,D_ ~ 1 × 10^8^ cm^−2^, while ρ_B_~ 8 × 10^7^ cm^−2^.

High aspect ratio and high refractive indices of the materials used result in good waveguiding properties along the axes of our NWs. Since GaAs has bandgap wavelength at around 870 nm, material absorption at shorter wavelengths will lead to losses along the propagation, resulting in real component of the transverse wavevector inside NW. This means that the light coupled to these modes will transversely leak out, and they are correspondingly called leaky waveguide modes. Their electric field is mainly confined outside NW, contrary to the guided modes. Depending on the symmetry of the incoming electric field, for the TM or TE polarization, these modes can be HE_mn_ - magnetoelectric (TM_mn_-like), or EH_mn_ - electromagnetic (TE_mn_-like), respectively. Here m is the m-th order of the corresponding Bessel and Hankel functions that are the solutions of a leaky guided wave in the infinitively long dielectric cylinder, and n corresponds to the n-th zero of these functions. In the case of finite NWs, and the illumination parallel to the NW axis, light scattering at the end facets can provide for the additional coupling where the leaky modes become guided^5^. Such modes result in resonant enhanced absorption that has HE_mn_ or EH_mn_ symmetry along NW, and we first numerically investigate their excitation in our samples by simulating the absorption cross section (see Methods and Supporting Information).

**Figure 3:**
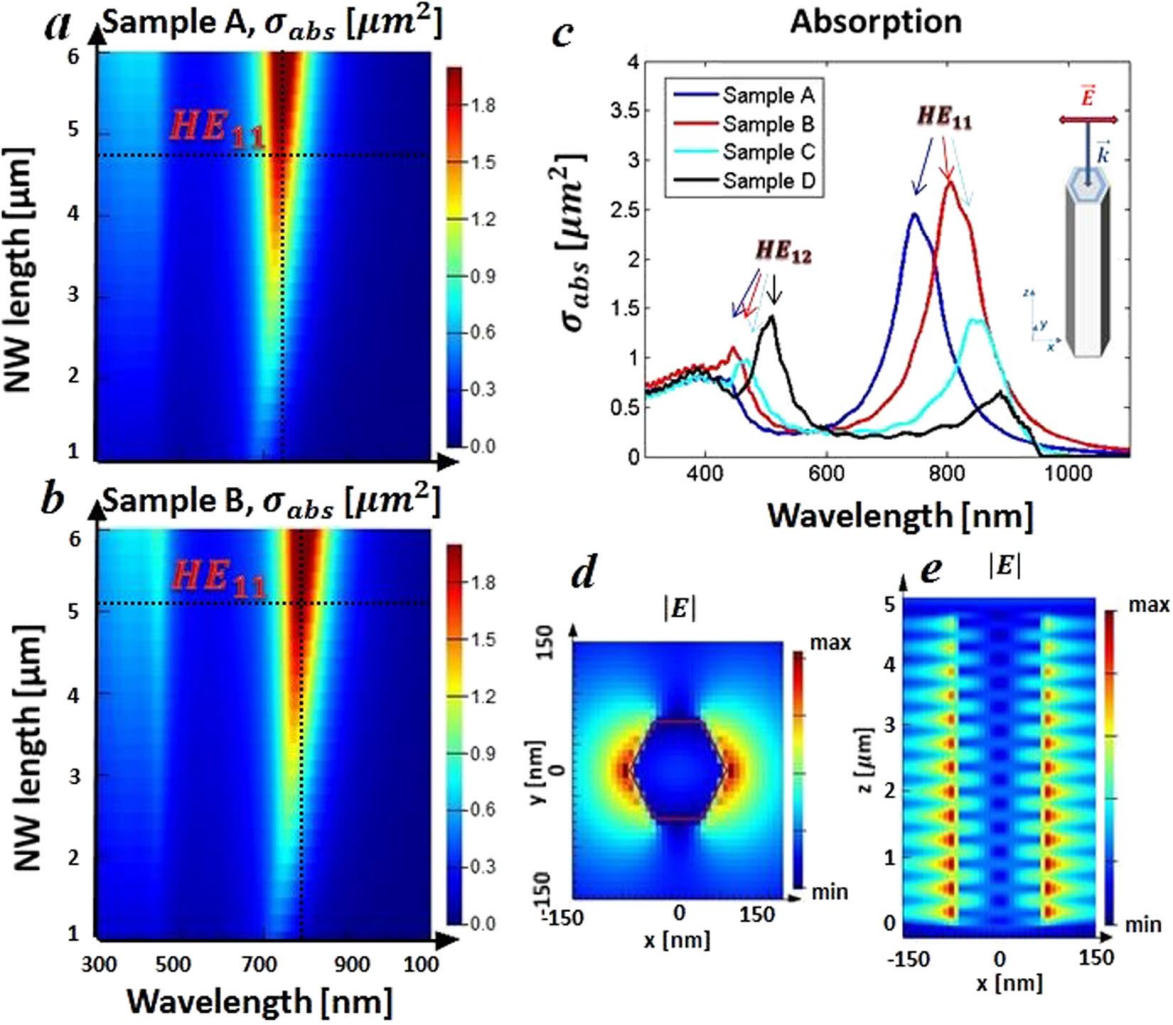
(**a**) Sample A: absorption cross section dependence on L. (**b**) Sample B: absorption cross section dependence on L. (**c**) Absorption cross section spectra for samples A–D calculated with the geometric parameters of Table 1. (**d**) x-y cross section: electric field confinement at HE_11_ wavelength (Sample A is used as an example). (**e**) x-z cross section: electric field confinement at HE_11_ wavelength (Sample A is used as an example).

In Fig. 3 we show the absorption dependence on NW length, using the geometric cross section parameters from the fabrication of Samples A-D. Fig. 3a clearly shows that HE_11_ mode for the sample A is excited at around 730 nm, while for the sample B HE_11_ resonance moves to around 790 nm (Fig. 3b). This is in agreement with theory because the increase of an effective waveguide diameter red-shifts the resonance of the modes. It is also evident from Fig. 3a,b that the NW length mainly affects the absorption efficiency while the influence on the wavelength of the resonant mode is very small. In Fig. 3c the calculated absorption cross sections for all four samples are shown. Again, the red shift due to the increasing of diameter D from sample A to sample D is clear. Moreover, the geometric parameters of sample D would lead to HE_11_ resonance at λ > 870 nm; however, in this range both GaAs and AlGaAs are transparent, therefore sample D, and partially sample C do not show such high absorption for the HE_11_ mode. At around 440–500 nm another peak due to the coupling to HE_12_ mode arises in all four samples, but it has much lower intensity. In Fig. 3d we see that the field confinement corresponds to the symmetry of HE_11_ mode in the x-y cross section, while the mode is guided along the wire as shown in the x-z cross section (Fig. 3e).

**Figure 4:**
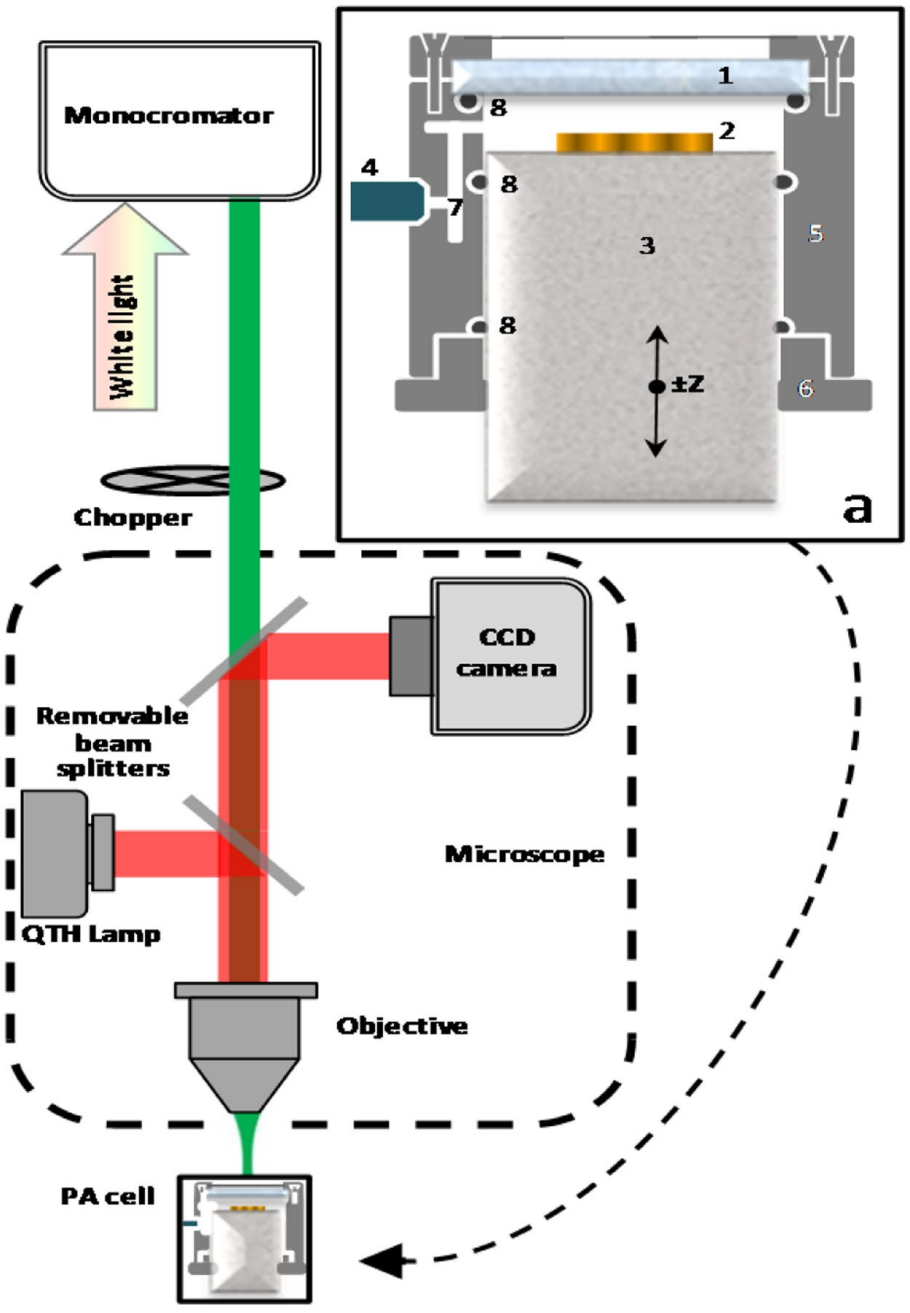
Experimental set-up of the photo-acoustic measurement. Inset (**a**): Variable volume photoacoustic cell, 1-quartz window, 2-sample, 3-quartz cylinder, 4-microphone, 5-inox cell body, 6-threaded flange, 7-sound labyrinth, 8-O-ring.

We then experimentally characterize our samples by measuring their absorption by means of the photo-acoustic (PA) technique. PA is a type of photothermal techniques, where absorption of incoming light beam leads to a non-radiative de-excitation process which generates heat, thus changing the thermal state of the sample^30, 31^. If the intensity of the light is modulated in time, a sample in an air-tight chamber heats up and cools down in a cycle and, not having time to expand and contract, corresponding changes in pressure arise. These pressure changes produce an acoustic signal which is then directly converted into an electrical signal by a sensitive microphone. The microphone receives the signal from the sample through the small diameter tunnel (labyrinth) so that the light cannot impinge the microphone directly, thus largely reducing the effects of the scattered light (Fig. 4a). By this way we can directly measure the near field absorption, and possibly catch the effects that would be otherwise hidden with other scattering dependent techniques. PA technique has been widely used for the imaging of plasmonic nanoparticles for biomedical applications, dealing with the localized surface plasmons^32, 33^. Moreover, it has been recently applied to study NWs^28, 34–37^; in ref. 38 we have applied PA technique to demonstrate circular dichroism in GaAs-based NWs asymmetrically hybridized with Au, where three out of six sidewalls were covered with Au, thus leading to the symmetry breaking. However, to our knowledge no work has been performed on investigation of resonant absorption in vertical semiconductor NWs which is essential for the development of NW arrays for NW-based photovoltaics^11^ and wavelength selective photodetectors^6^. We therefore apply the set-up shown in Fig. 4 to measure the absorption in the spectral range from 300 nm to 1100 nm, where we expect the excitation of HE_11_ and HE_12_ modes in all the samples (see Methods).

**Figure 5:**
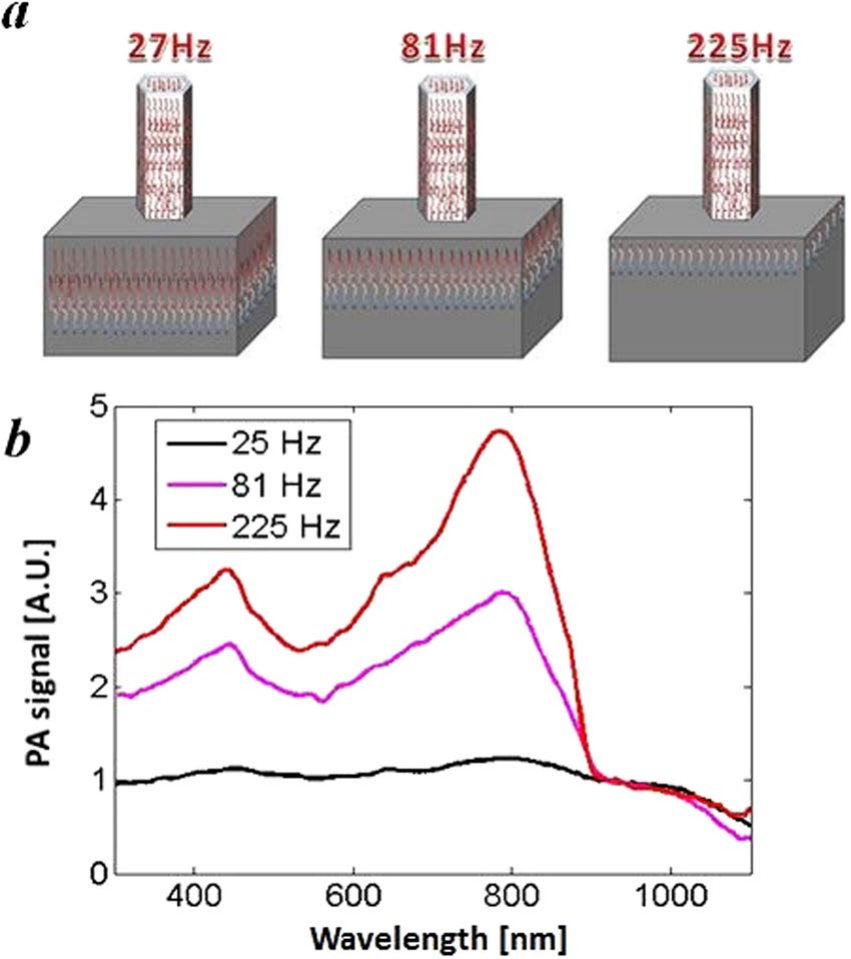
(**a**) Schematic of a different surface / NW contributions for different frequencies: at the lowest 27 Hz the PA signal mainly originates from Si substrate, while at the highest 225 Hz it mainly originates from NW absorption. (**b**) PA spectra for three different frequencies that correspond to the different contribution of the sample surface for sample B. At the highest frequency 225 Hz we observe the resonant peak corresponding to the excitation of HE11 mode.

In photothermal techniques the thermal diffusion length is inversely proportional to the square of the modulation frequency^39, 40^. This means that with lower frequencies thermal signal takes much higher non-resonant contribution of Si substrate and parasitic (Al)GaAs growth. With the frequency increasing the contribution of the NW surface is higher, so we can see the resonant response which corresponds to the excitation of the discrete modes, as it is depicted in Fig. 5a – for the low frequency (27 Hz) most of the absorption comes from the heat generated in Si substrate (red to blue arrows), while for the highest frequency of 225 Hz the signal originates mainly from the NW absorption. The absorption spectra for three frequencies of Sample B is shown in Fig. 5b. The spectra are normalized to the spectra of the substrate (Supporting information, Figs S5–7). For the lowest frequency of 27 Hz we see the highest contribution of Si substrate, therefore the Si bandgap, and the expected resonant absorption is hidden by this contribution. Then, at 81 Hz, we start to observe a resonant behavior, with the increased contribution of the parasitic GaAs growth and NWs (GaAs bandgap is seen at 870 nm). Finally, at high frequency of 225 Hz we clearly observe the resonant HE_11_ peak which corresponds to the highest contribution of the NW surface that supports this resonance. Similar influence of frequency on the NW PA signal was recently reported for GaAsBi/GaAs core-shell NWs grown on Si^28^. However, no resonant features were observed most probably due to size non-uniformity of the investigated NW ensembles.

The contributions of the Si substrate and parasitic growth and NWs on the PA signal were investigated in detail by analyzing reference samples (Supporting information, Fig. S4). We conclude that at high frequency of 225 Hz the non-resonant signal arises mainly from the Si substrate while the contribution of the parasitic layer is very small (Fig. S6). More importantly, the intensity of the non-resonant signal is small compared to the resonant signal arising from the NWs. Therefore, our useful PA signal coming from the resonant response is stable at this frequency, and we continue our measurements keeping f_mod_ = 225 Hz.

**Figure 6:**
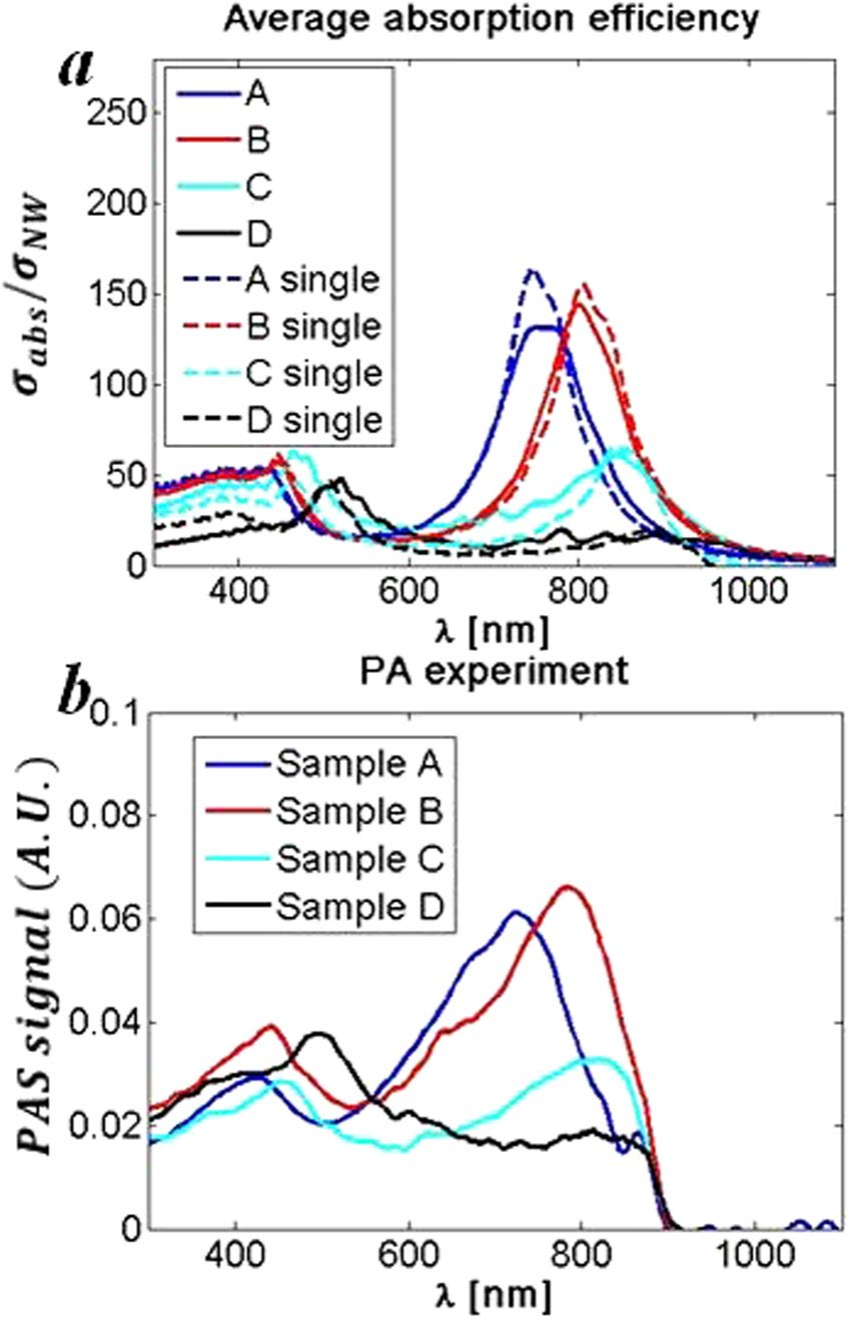
(**a**) Simulated average absorption efficiency including the actual NW size distribution for Samples A-D. Single NW simulations included for the sake of comparison. (**b**) PA spectra for Samples A-D.

In Fig. 6 we show the simulated (Fig. 6a) and measured (Fig. 6b) spectra of our samples. We have taken into account the actual NW size distributions of the investigated samples (Table 1) by incorporating a random Gaussian distribution in the simulation; therefore, Fig. 6a shows an average absorption for this distribution generated in L-D parameter space. The absorption efficiency is defined as σ_abs_/σ_NW_, where σ_NW_ = D^2^π/4 is the NW projected area. We see that these efficiencies at HE_11_ resonances are as high as 130 and 150, for Sample A and Sample B, respectively, while for bulk planar materials these are always ≤1. We see that the experiment is in great agreement with theory – namely, the positions of H_11_ and H_12_ modes almost perfectly match to those of PA signals. However, the NW size distributions cannot fully explain the observed PA peak broadening (dashed lines in Fig. 6a show the simulation for a single NW without the actual size distribution). The other factors that may contribute the peak broadening include the interaction between adjacent NWs as well as slightly tapered shape of the NWs. As shown in the Supporting information (Fig. S2), the degree of tapering increases with increasing NW thickness from Sample A to Sample D. Therefore, we would expect to see significant differences in the peak widths of different samples. Since this is not the case (Fig. 6b), we can exclude the possible influence of NW tapering on the peak broadening.

**Figure 7:**
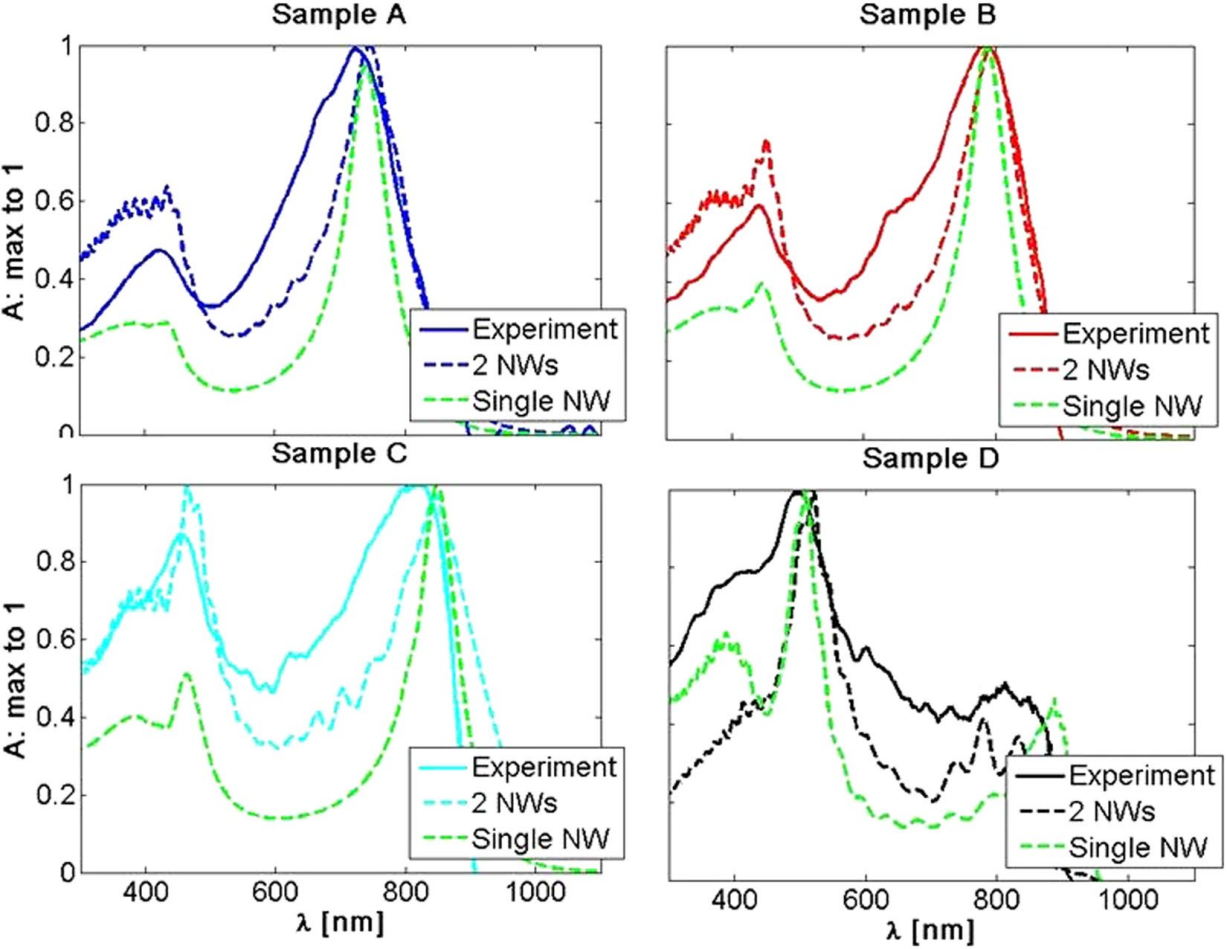
FDTD simulations for two NWs compared with PA technique and single NW spectra, and normalized to 1.

To investigate the collective response of the closely spaced NWs, we have performed FDTD simulations to account for their interaction. The nearest neighbor statistics from top SEM images (Fig. S8) is shown in the Supporting information (Fig. S9). We repeated the simulations for two NWs using these statistics and keeping their other geometric parameters constant (Table 1). We apply again PML in all directions and simulate absorption cross section of the NW. These results along with PA experiment results are then normalized to 1 to show the shape differences, Fig. 7. Results for the single NW are shown as green dashed lines. We see that this two NW simulation fits well with PA spectra for all four samples and that it leads to the broadening with respect to the single NW resonance; therefore, changes in distances between neighbor NWs are one of the reasons for the broadening of the spectra. However, the collective effects of such disordered NW arrays are in general difficult to simulate precisely due to the memory and time consuming simulation sizes. PA technique offers a possibility to directly investigate the absorption properties and therefore catch these effects, which is a big advantage over other techniques. Furthermore, a single NW theory is suitable to predict the behavior of sparse NW arrays^4^; therefore, we firmly believe that PA can be efficiently used to measure the resonant absorption also in ordered NW arrays.

In order to compare PA results with a conventionally used reflection technique, we report on the reflection spectra measurements (Figs S10 and 11) of the samples at normal incidence. In the Supporting information we show that the results are again in agreement with PA technique (Fig. S11) - the positions of reflection dips almost perfectly coincide with the absorption maxima of PA signal. Nevertheless, we point out that for an absolute measurement of the absorption from the reflection, a calibration mirror and an integrating sphere detector would be required to account for scattered fields. Moreover, such characterization usually requires the invasive techniques as the removal of NWs from the substrate^41^. On the other hand, the method proposed allows for direct, scattering-insensitive absorption measurement.

## Discussion

Our results have demonstrated that PA technique can be employed as a simple and reliable characterization technique that measures the enhancement in the absorption due to the excitation of photonic modes in GaAs-AlGaAs-GaAs core-shell-supershell NWs. The measurements evidence a highly efficient absorption enhancement under proper resonant wavelength for H_11_ mode and it is in great agreement with numerical simulations. Since this technique directly measures the absorption, no post-processing of the signal is needed like in transmission/reflection and other similar techniques. We also show that the response of our samples is very stable with respect to the fabrication margins of the parameters D and L; we further simulate the response of two NWs the distance of which is taken from the nearest neighbor statistics. We believe that this absence of the uniformity of the distance between adjacent NWs is the primary reason for the broadening of the resonances, but they remain pronounced. Present efforts are dedicated to specific improvements of our stable and low-cost PA set-up to allow for the use of both qualitative and quantitative characterization of samples to be used for the realization of many applications of NW such as NW lasers and chirality applications of GaAs-based NWs asymmetrically covered with Au.

## Methods

### NW fabrication

The GaAs core was first grown by self-catalyzed growth mode. Then the Ga catalyst droplet was consumed in As2-flux in order to terminate axial growth. The Al_0.3_Ga_0.7_As shell and GaAs supershell were then grown around the NW core using growth conditions that promote radial growth. The details of the Si/SiOx pattern fabrication and NW growth are explained in more detail in^29^ and in Supporting information (1-2), which includes also the description of the shell layer growth and characterization of the coaxial heterostructure (Fig. S2-S3 and Table S1).

### Numerical calculations

We have used Lumerical Finite Difference Time Domain (FDTD) solver to simulate the absorption cross section spectra of a single GaAs-AlGaAs-GaAs NW, by applying perfectly matched layers (PML) in all directions. A single NW is excited by total-field scattered-field source (TFSF) that is suitable for simulations of the absorption cross section in a box surrounding the NW (Supporting information 6).

### PA absorption

The samples are shined from the air side by a Xenon arc lamp source followed by a monochromator, which provides the spectral range from 300 nm to 1100 nm. This source is modulated by a mechanical chopper whose frequency choice sets the contribution of the NW surface to the overall absorption. The output of the microphone pre-amplifier (PA) is connected with the input of the lock-in amplifier and analyses the signal with respect to the modulated frequency of the light source. The main element of the cell is a movable transparent quartz cylinder, with two optically polished faces, which offers some advantages: its movement along the cylinder axis direction changes the distance between the sample surface and the window, so that we can choose the optimum volume of the cell. All the samples are shined under a normal incidence, but our set-up is adaptable also for incidence angle and polarization scan.

## Electronic supplementary material

Photo-acoustic spectroscopy revealing resonant absorption of self-assembled GaAs-based nanowires – SUPPORTING INFORMATION
