# Supplementary material for: Photo-acoustic spectroscopy revealing resonant absorption of self-assembled GaAs-based nanowires
Source: Sci Rep. 2017 Jun 6;7:2833. doi: 10.1038/s41598-017-02839-1 (PMC5460253; doi:10.1038/s41598-017-02839-1)
Supplement: Supplementary file 1 — Photo-acoustic spectroscopy revealing resonant absorption of self-assembled GaAs-based nanowires – SUPPORTING INFORMATION [file 41598_2017_2839_MOESM1_ESM.pdf]

Photo-acoustic spectroscopy revealing resonant absorption of self-assembled GaAs-based nanowires – SUPPORTING INFORMATION

Grigore Leahu[a], Emilija Petronijevic[a], Alessandro Belardini[a], Marco Centini[a], Roberto Li Voti[a], Teemu Hakkarainen[b], Eero Koivusalo[b], Mircea Guina[b], Concita Sibilia[a]

a. Dipartimento di Scienze di Base ed Applicate per l'Ingegneria, Sapienza Università di Roma, A. Scarpa 16, 00161 Rome, Italy

b. Optoelectronics Research Centre, Tampere University of Technology, Korkeakoulunkatu 3, 33720 Tampere, Finland

1. Nanowire fabrication

NWs were grown by molecular beam epitaxy on p-Si(111) wafers using lithography-free Si/SiOx patterns for defining the nucleation sites. The patterns were defined using droplet epitaxy of GaAs nanocrystals, spontaneous oxidation, and thermal annealing. This technique is described in detail in [1], where it is shown that the NW density and diameter can be controlled by the GaAs nanocrystal density and diameter, respectively. The NW samples investigated in this letter were grown using templates having GaAs nanocrystal diameter of 47 nm and density of 1.7×10^{8} cm^{-2}.

The NW core was grown by self-catalyzed method at 640°C using Ga flux corresponding to a planar GaAs(100) calibrated growth rate of 0.3 ML/s and As_2/Ga beam equivalent pressure (BEP) ratio of 9. The growth duration was 1h. Consequently, the Ga droplet was consumed in As_2 flux in order to terminate axial growth. The crystalline structure of the GaAs core was studied in [2], where it was shown that the NWs are predominantly defect free zincblende while around 150 nm long wurtzite segments are formed at the tips of the NWs during crystallization of the Ga droplets. The $Al_{0.3}Ga_{0.7}As$ shell was grown using BEP ratio of 15 and 0.45 ML/s growth rate. The GaAs supershell was grown BEP ratio of 17 and 0.3 ML/s growth rate. The growth temperature for the $Al_{0.3}Ga_{0.7}As$ shell and GaAs supershell layers was 640°C for sample A and B, and 555°C for samples C and D. The thickness of the shell layers was varied in samples A-D, while the core dimensions were kept constant.

2. Determination of nanowire dimensions

Cross-sectional SEM images shown in Fig. S1 illustrate the remarkable size uniformity of the GaAs-AlGaAs-GaAs core-shell-supershell NWs obtained by self-catalyzed growth on lithography-free Si/SiOx patterns. More detailed analysis of the nanowire dimensions, was performed by measuring length L and diameter D for >20 NWs per sample in order the determine the average value and standard deviation of the distribution. Furthermore, it was observed that the NWs have some degree of inverse tapering due to flux shadowing effects; the diameter at the top of the NW (D_{top}) is larger than the diameter at the bottom of the NW (D_{bottom}). This is most clearly visible in Sample D with thickest shell layers (Fig. S1(d)). The degree of tapering for Samples A-D is

illustrated in Fig. S2. It should be noted that the average diameter *D* corresponds to the NW diameter in the middle of the NW.

The average thicknesses of the shell layers was estimated from the nominal thicknesses based on geometrical consideration of the incident flux on the NW sidewalls [3] and the increase of the overall diameter during the shell growth. For this purpose we fabricated a core-only reference sample (*L*=4180+/-90nm, *D*=130+/-6nm). In case of Samples C and D, the estimated shell thicknesses were similar to the nominal ones. However, the shell layer thicknesses in Samples A and B were smaller than the nominal values because of the high shell growth temperature, which induced significant axial growth. The length and diameter statistics and estimated shell layer thicknesses of samples A-D are shown in Table S1.

The coaxial heterostructure was further investigated by cross-sectional transmission electron microscopy (TEM). A cross-sectional TEM micrograph of sample D is presented in Figure S3 along with EDS maps showing distributions of As, Ga, and Al atoms. The photoluminescence properties of the sample D were reported in [1], where it was shown that the shell structure gives rise to a 1000-fold increase of the photoluminescence intensity. Furthermore, room temperature time-resolved photoluminescence measurement of sample D results in biexponential decay with time constants of 1.0 ns and 3.0 ns. The TEM and PL results indicate successful growth of high quality shell structure.

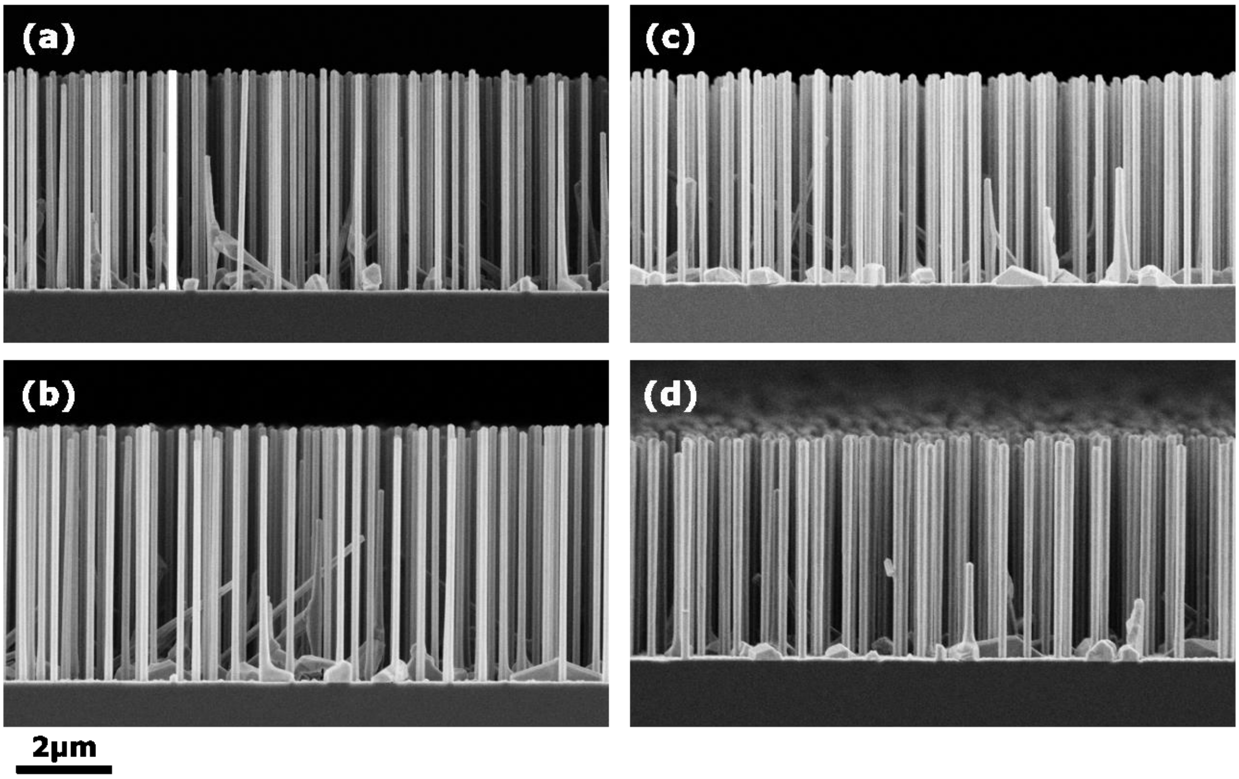

Figure S1: Cross-sectional SEM images of NW Samples A (a), B (b), C (c), and D (d).

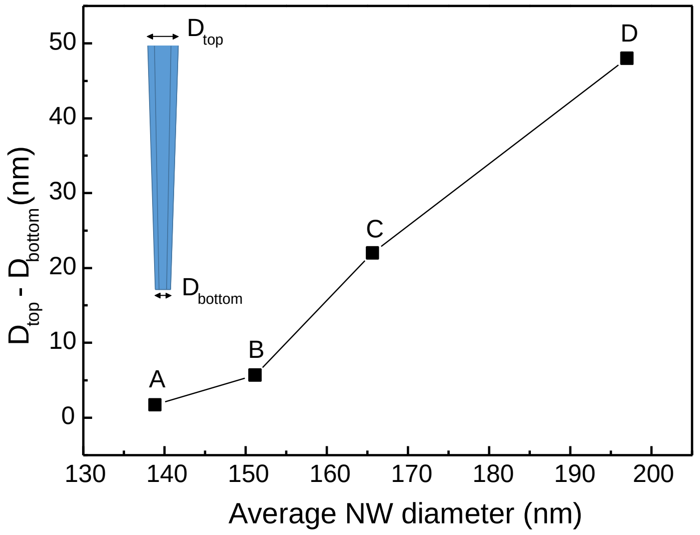

Figure S2: Degree of tapering for NW samples A-D.

			Shell thickness [nm]	
Sample	Length *L* [nm]	Diameter *D* [nm]	AlGaAs	GaAs
A	4750+/-34	138+/-5	3.5	0.7
B	5190+/-64	151+/-5	8.6	1.7
C	4600+/-52	165+/-6	11.7	5.8
D	4690+/-80	197+/-9	27.7	5.5

Table S1: NW dimensions and shell layer thicknesses for Samples A-D.

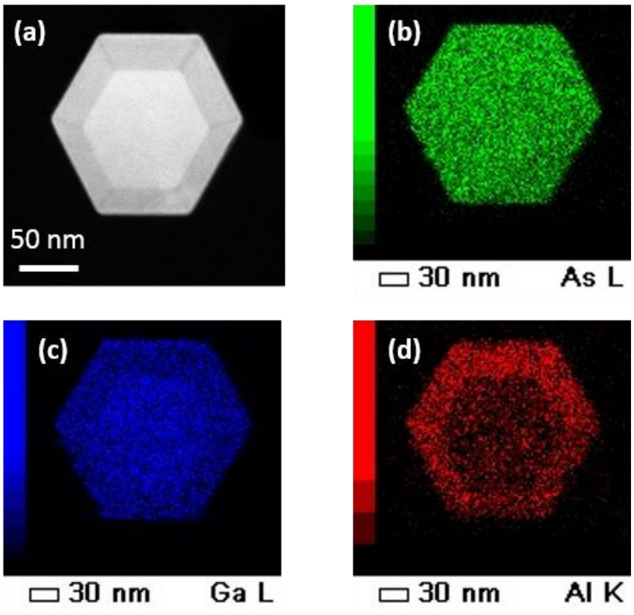

Figure S3: Cross-sectional dark-field TEM micrograph of Sample D (a), and EDS maps showing distribution of As (b), Ga (c), and Al atoms (d).

3. Photoacoustic response of Si substrate and parasitic growth

Two reference samples were used for assessing the relative contributions of NWs, Si substrate, and parasitic growth on the PA response. First reference sample was a p-Si(111) wafer, similar to what was used as a substrate for the NW growth. The second reference sample, intended for investigating the contribution of parasitic growth, was fabricated be removing the NWs from Sample C in ultrasonic bath. SEM pictures of Sample C before and after the sonication are shown in Figure S4.

PA spectra of the reference samples collected using different frequencies are shown in Figures S5-7 in comparison with NW sample B.

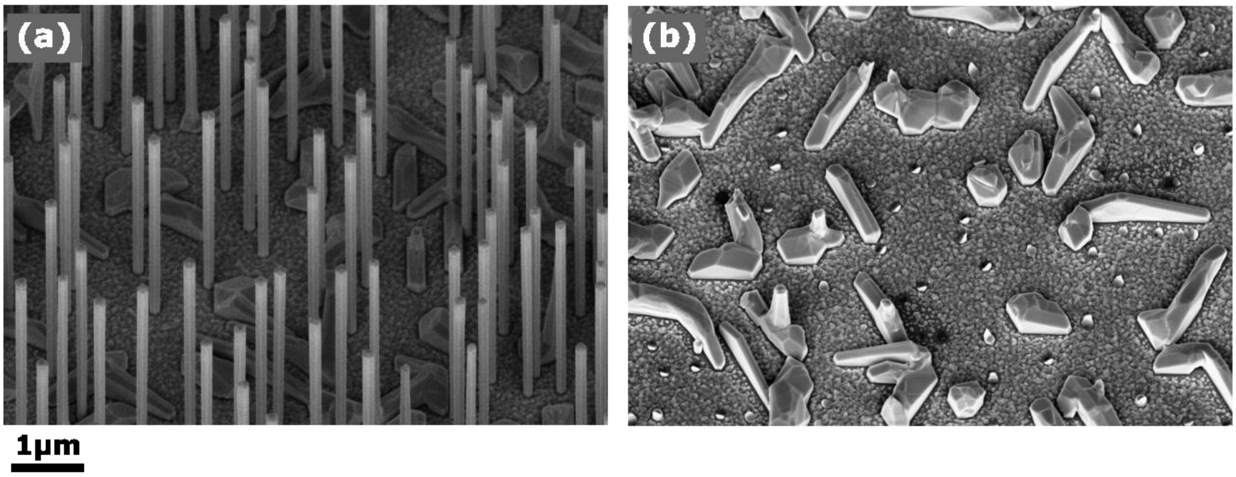

Figure S4: Tilted (30°) SEM image of sample C before (a) and after (b) removal of the NWs in ultrasonic bath.

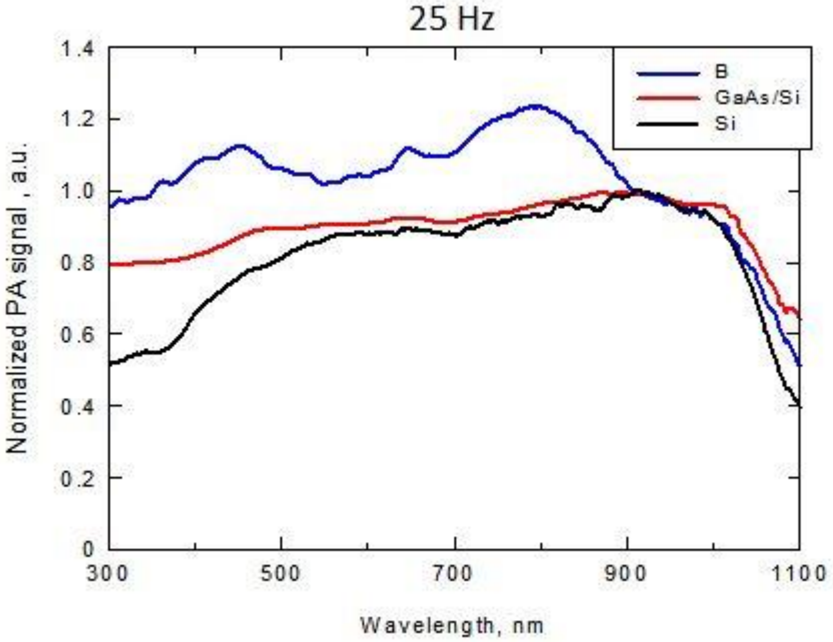

Figure S5: Absorption spectra of GaAs/Si and Si substrates at 25Hz compared with sample B.

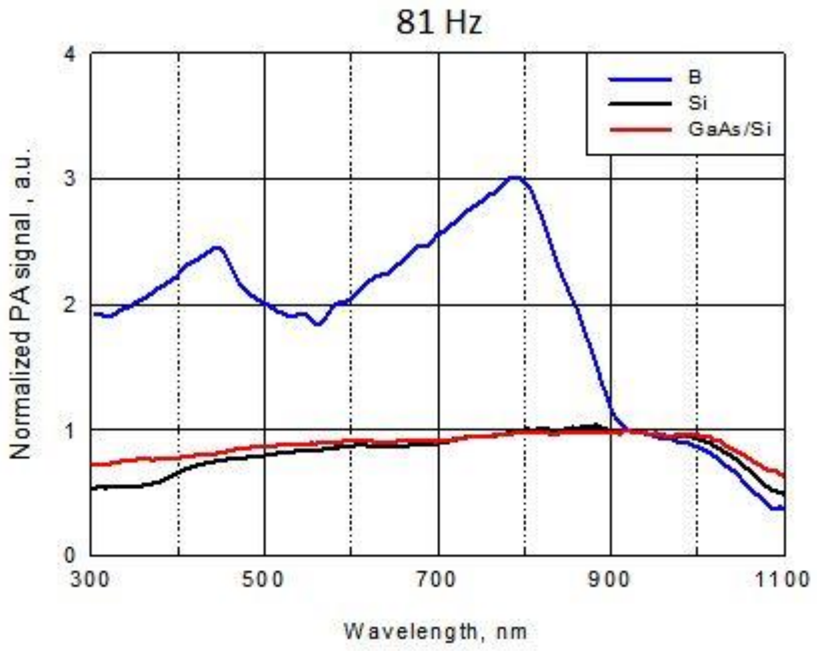

Figure S6: Absorption spectra of GaAs/Si and Si substrates at 81Hz compared with sample B.

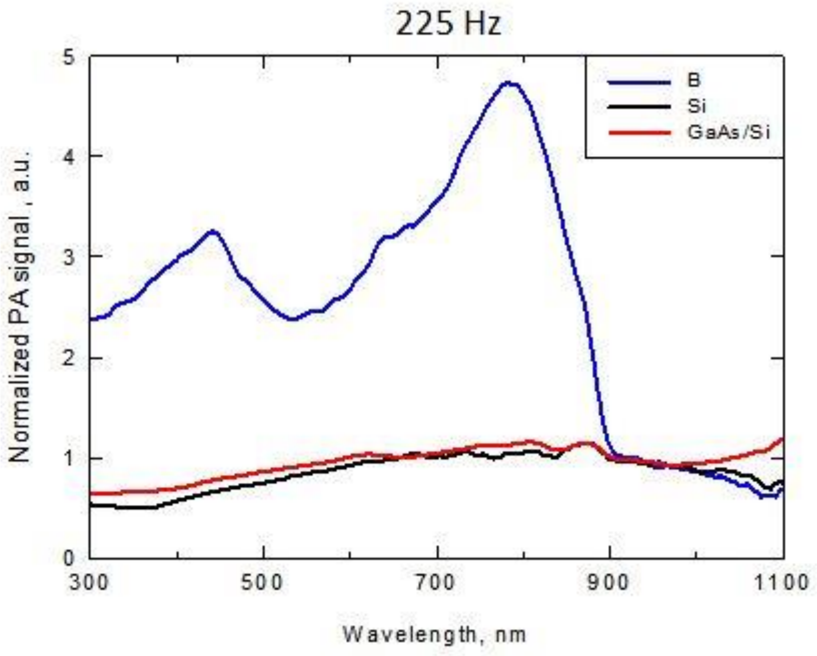

Figure S7: Absorption spectra of GaAs/Si and Si substrates at 225Hz compared with sample B.

4. Nearest neighbor statistics

The nearest neighbor distribution for NW Samples A-D were calculated from top-view SEM pictures shown in Figure S8. The histogram presentations of the distributions along with Gaussian fitting functions are shown in Figure S9. The nearest neighbor distributions of Samples A, C, and D

are very similar, centering at around 500-600 nm with standard deviation of ~180 nm. Sample B has larger nearest neighbor distance due to slightly lower NW density compared with the other samples.

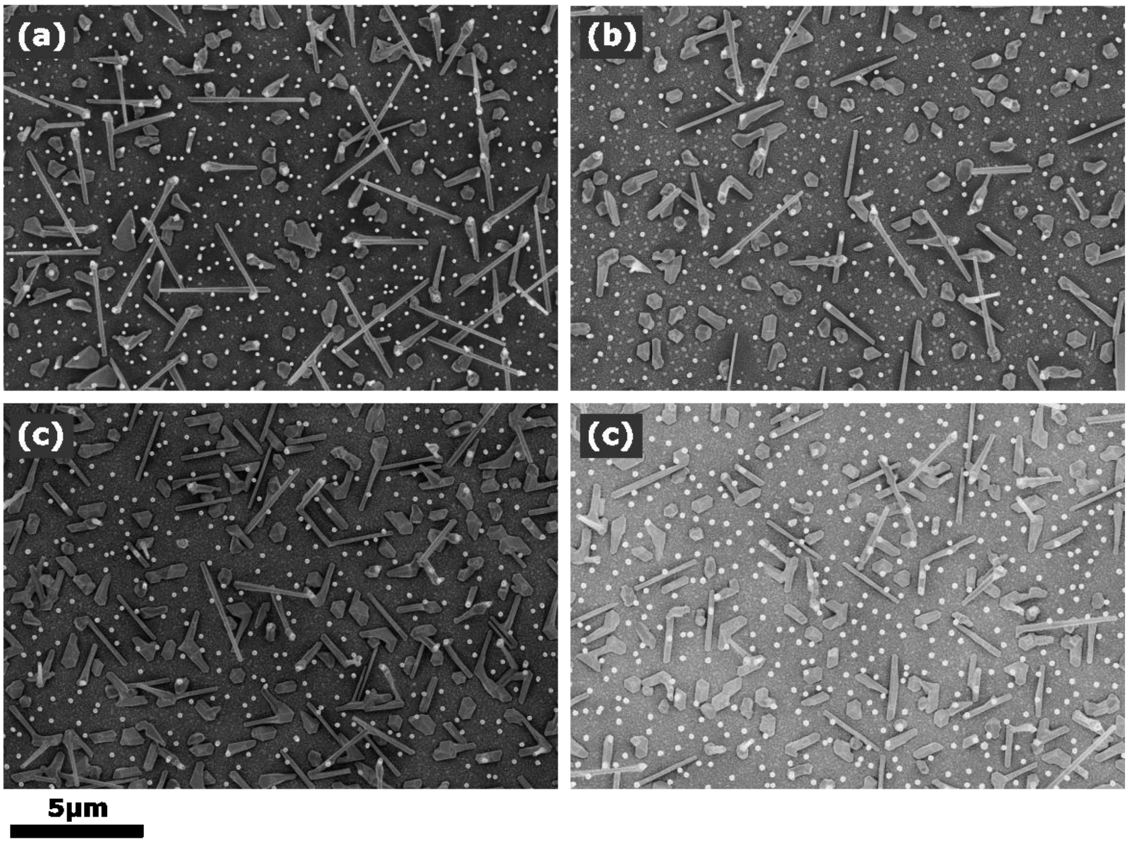

Figure S8: Top-view SEM images of NW Samples A (a), B (b), C (c), and D (d).

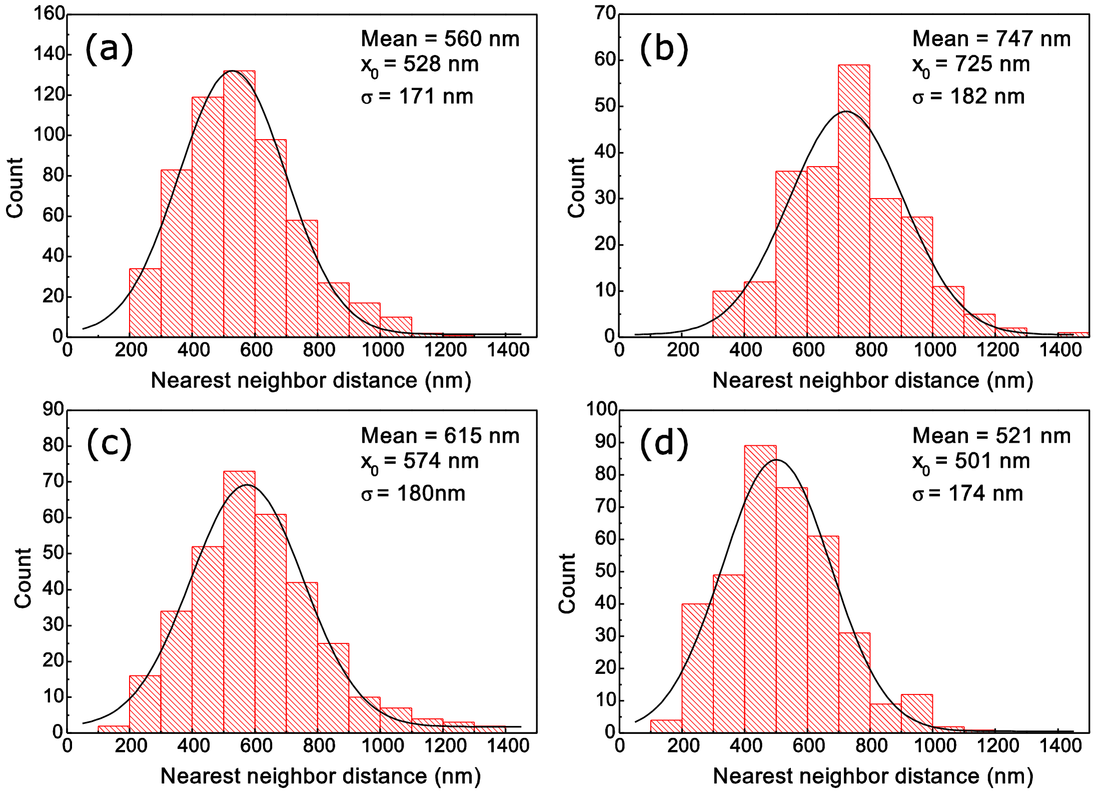

Figure S9: Nearest neighbor distance statistics for Samples A (a), B (b), C (c), and D (d). Each histogram includes the mean value of the data and center (x_0) and standard deviation (σ) of the Gaussian fitting function.

5. Reflection spectra measurements

In the manuscript we have used the photo-acoustic technique to study absorption properties of semiconductor nanowires. In order to confirm the validity of our results, we used conventional characterization by means of reflection measurements. The reflection set-up is shown in Figure S10. Xenon arc lamp (ORIEL) is followed by a monochromator which allows for the wavelength span of 300-1100nm. The light is then directed to a beam splitter so that it impinges under normal incidence on the samples. The beam splitter directs the reflected light to a photo-diode that measures the reflection spectra.

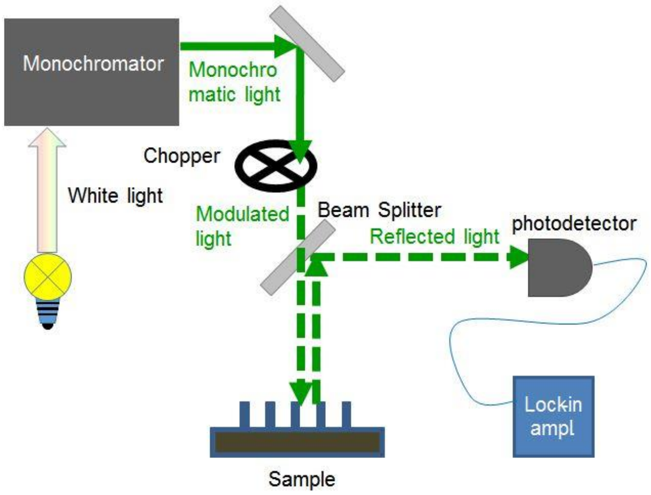

Figure S10: The set-up of the reflection measurement.

In Figure S11 we show both PA and reflection measurement results. Reflection is normalized to the reflection spectra of Ag mirror (flat reflectance of 97%). We see that the positions of PA peaks, that correspond to the excitations of the modes predicted by the calculations, coincide perfectly with the positions of reflection dips for both the samples. However, there is a broadening of reflection dips due to the scattering. Moreover, the reflection measurements always need to be calibrated, therefore the post-processing is required to obtain the useful signal. This leads to additional steps that become particularly complicated when the oblique incidence is investigated. On the other hand, PA technique offers a scattering-independent direct measurements of absorption, and our PA set-up has been successfully used to investigate properties of the samples under oblique incidence.

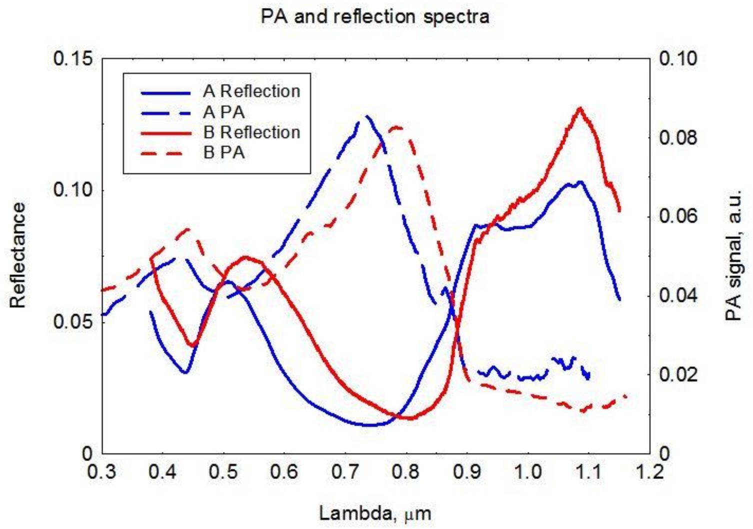

Figure S11: Reflection and PA spectra for samples A and B.

6. Numerical calculations in Lumerical, Inc

All numerical calculations have been performed using a commercial-grade simulator based on the 3D Finite Difference Time Domain (FDTD) method in Lumerical [4]. FDTD solves Maxwell's equations in complex geometries on a discrete spatial and temporal grid. Simulation region around a vertical NW was defined by perfectly matched layers (PML) in all directions, similar to the schematic in [5]. To ensure the stability and reliability of calculations, PML were at least half the maximum wavelength far from the NW surfaces, and the computational mesh around the NW was 1nm in x and y directions, and 10nm in z-directions (Fig. 1a). NW was excited by a total-field scattered source (TFSF) which is used to discriminate the computational region with the total field from the one with the scattered field. The absorption cross section spectra was then calculated by surrounding the NW with a box in the total field region. The same approach was applied for size-distribution averaging and two NWs simulation. To simulate the electrical field confinement along the NW and the cross-sectional one, field profile monitors were placed in *xz* and *xy* planes, respectively.

References

[1] Hakkarainen, T. V., Schramm, A., Mäkelä, J., Laukkanen, P. & Guina, M. Lithography-Free Oxide Patterns as Templates for Self-Catalyzed Growth of Highly Uniform GaAs Nanowires on Si(111). *Nanotechnology* **26**, 275301 (2016).

[2] Koivusalo, E., Hakkarainen, T. V. & Guina, M. Structural Investigation of Uniform Ensembles of Self-Catalyzed GaAs Nanowires Fabricated by a Lithography-Free Technique. *Nanoscale Res. Lett.* **12**, 192 (2017).

[3] Rudolph, D. et al. Spontaneous alloy composition ordering in GaAs-AlGaAs core-shell nanowires. *Nano Lett.* **13**, 1522 (2013).

[4] Lumerical Solutions, Inc. http://www.lumerical.com/tcad-products/fdtd/

[5] Henneghien, A-L., Gayral, B., Désières, Y. & Gérard, J-M. Simulation of waveguiding and emitting properties of semiconductor nanowires with hexagonal or circular sections. *J. Opt. Soc. Am. B* **26**, 2396-2403 (2009).
